# Annotations

**Published:** 1896-07-18

**Authors:** 


### ANNOTATIONS.

### Legalised Cruelty.

WE are all apt to believe that England is entitled to take credit for its kindness to animals. This feeling arises from the belief that cruelty cannot be habitually practised without punishment, and so the public come to believe that continuous cruelty to animals is a thing of the past. The proceedings before the Colchester magistrates on the 7th inst., where it was proved that all the English railways habitually practice gross cruelty to cattle in transit has, therefore, created a feeling of painful surprise. The grateful recognition of all humane persons is due to the *Times* newspaper for the publicity it has given to the case, and for its strenuous appeal for the abolition of the existing system. The remedy rests entirely with the general managers of the British railways, who, by the issue of one circular, could get rid of the cruelty for all time by providing that the cattle shall for the future be carried only in trucks fitted with movable compartments, so that each animal may have the equivalent of a separate stall. Such a reform would, in the long run, prove a saving to the railway companies; the cattle would be protected from injury, and their value would thereby be increased, and the servants of the company would be relieved from a particularly odious duty. Is it too much to hope that the appeal now made to the railway managers, chairmen, and directors will find a ready response, and that England may be freed from the hideous cruelty attaching to the present system of removing cattle by railway from the markets to the butchers to whom they have been consigned? Our appeal must necessarily lie with the railway companies first, but, failing this, there can be no doubt from the attention which this case has already received that Parliament will speedily interfere, should such a course be necessary—which we do not for one moment believe, now that public attention has been definitely directed to the gross cruelties connected with the present system.

### Milk Dealers and the Law.

OF all things milk is the easiest to adulterate, and it would seem that by a little ingenuity, together with some knowledge of the law, there can be few forms of adulteration from punishment for which it should be so easy to escape. The outcome of a recent trial is to show in a marked manner how easily a written warranty from the country milk producer may be made to protect the London dealer, while entailing no risk upon the producer, and may thus render futile all efforts to punish the adulterator. We do not suggest that in the particular case which came before the court there was any collusion of the kind. The probabilities are that it was one of the ordinary cases, in which the question whether or not there is adulteration hinges on the standard of purity accepted by the analyst. But the defence set up was so successful, and, from a legal point of view, so perfect, that it is sure to be used for the protection of fraud in the future. A public institution got its milk from a dairy company, and this milk being analysed, as delivered, was pronounced to contain added water. But the dairy company were able to prove that they supplied the milk as it was received, and they put in a written guarantee that they had purchased the milk as the same in nature, substance, and quality as that demanded of them, and had received from the farmer a written guarantee to that effect. The case against the dairy was therefore dismissed. The sanitary inspector then charged the farmer with giving a false warranty, but both the dairy and the farm were outside the magistrate's district, and he declined to hear the case. Application was then made to the Queen's Bench Division for a rule to compel him to do so. This, however, was refused by the judge, who held that, however good the certificate of the public analyst was as against the dairy company who supplied the milk, the analysis being, in fact, of their milk as delivered, it could not be put in as evidence against the farmer; and that, there being no evidence of adulteration of the milk as delivered by the farmer to the dairy company, the action must fail. It is sufficiently obvious, then, that a milk dealer, receiving milk from perhaps half-a-dozen farms, can protect himself by a series of warranties; for so long as the farmers send good milk no action would lie against them, even if by good luck the inspectors were able to sample the right milk in transit, while, unless the dealer could be caught in the act of adding the water, the written warranty would be his protection against any action on account of water found in the milk which he supplied. Clearly this question of warranty will require careful consideration whenever the legislation on this subject is overhauled.

### Physic for Bad Temper.

WHEN boys become stupid, sulky, and ill-tempered some schoolmasters cane them; others, with a wider knowledge of the relations between mind and matter, give a dose of castor oil—and not uncommonly with effects most salutary. Dr. Lauder Brunton applies the same principle to patients of greater age, and seeks by medicine to cure the irritability of temper which is so commonly associated with gout and heart disease. Writing in the *Practitioner*, he points out, what is well known to all who have seen much of short-tempered people, that explosions of temper which occur on very slight provocation are really due to a condition produced by an accumulation of small irritations which have gradually worked up the patient into a state of excitement which vents itself in an explosion quite out of proportion to its apparent cause. Continuous physical discomfort also has the same effect. But even without obvious discomfort the accumulation of abnormal substances, such as uric acid, may also produce irritability of temper. At any rate, in cases of gout, twenty grains of bicarbonate of potash with ten or twenty of bromide of potassium, taken when the feeling of irritability comes on, frequently soothes it; and if taken when some irritating occurrence has taken place, or some depressing news is heard, it appears to take away the sting of either. In some cases of cardiac disease also the bromide may be given with salicylate of soda with good success. All this is very true, although, to practitioners who have struggled long with the vagaries of restless patients, it may not appear very new. Dr. Brunton does, however, make a suggestion worth bearing in mind. Patients are sometimes seen whose appetites are spoiled, their digestion impaired, and their pleasure in life destroyed, not by any illness of their own, but by the constant fretfulness and irritability of some other member of their family. Here, if one can but get the other party to take these "temper powders," one may do better than by giving tonics to the patient.

